# Challenges faced when masking a single discoloured tooth - Part 1: aetiology and non-invasive management

**DOI:** 10.1038/s41415-025-8386-z

**Published:** 2025-06-27

**Authors:** May Aljanahi, Argwan Alhussin, Haitham Elbishari

**Affiliations:** 41415376109001 Hamdan Bin Mohammed College of Dental Medicine (HBMCDM), Mohammed Bin Rashid University of Medicine and Health Sciences (MBRU), Dubai Health, United Arab Emirates; School of Medicine, Dentistry and Biomedical Sciences, Queen´s University Belfast, Belfast, UK; Centre for Public Health, School of Medicine, Dentistry and Biomedical Sciences, Queen´s University Belfast, Belfast, UK; 41415376109002 Hamadan Bin Mohammed College of Dental Medicine (HBMCDM), Mohammed Bin Rashid University of Medicine and Health Sciences (MBRU), Dubai Health, United Arab Emirates; 41415376109003 Hamdan Bin Mohammed College of Dental Medicine (HBMCDM), Mohammed Bin Rashid University of Medicine and Health Sciences (MBRU), Dubai Health, United Arab Emirates; School of Medicine, Dentistry and Biomedical Sciences, Queen´s University Belfast, Belfast, UK; The Faculty of Biology, Medicine and Health, School of Medical Science, Division of Dentistry, University of Manchester, Manchester, England, UK

## Abstract

Encountering a single discoloured tooth is a common occurrence in dentistry and it poses a significant concern affecting both aesthetic appearance of natural teeth and patient confidence. Management of tooth discolouration involves a wide variety of options and requires specific protocols for both the clinician and patient to achieve an aesthetic result. One of the toughest challenges in restorative dentistry is being able to mimic natural teeth. This review is the first of two articles that will broadly discuss the aetiology of discolouration and the challenges faced when masking a single discoloured tooth. It will also examine various approaches, encompassing the conservative options, such as scaling, microabrasion, air abrasion, vital and non-vital tooth whitening, and resin infiltration. By integrating current and clinical evidence, this review aims to identify the causes of single tooth discolouration, highlight the challenges/variables faced when masking discoloured teeth and appraise possible minimally invasive procedures.

## Key points

- Calls attention to the aetiologies of a single discoloured tooth.
- Highlights minimally invasive management of a discoloured tooth, such as bleaching (vital and non-vital), abrasion, resin infiltration and direct composite veneers.
- Discusses treatment protocols of bleaching, abrasion, resin infiltration and direct composite veneers.

## Introduction

Tooth discolouration can influence an individual's confidence and behaviour in social situations.^1^ As a result, even a seemingly minor concern, such as a discoloured tooth, has become a considerable challenge, impacting not only the treatment process but also the overall management of the patient.^1^

Discoloured teeth have been found to significantly impact a patient's quality of life.^2^ Lately, there has been a rapid upsurge in aesthetics due to the increasing role of social media and the methods in which dental and other oral cosmetic procedures have been portrayed and perceived.^2^^,^^3^ The importance and evolution of aesthetics persisted in the twentieth century. Beall^4^ assessed how a full character judgement can be made based on one's appearance and the study concluded that photographs showing patients who had undergone major cosmetic dental treatment were ranked as being the most appealing.

This emphasises that aesthetics are influenced not only by the teeth themselves, but also by factors such as smile, personality, tooth colour, shape and size. In addition, an individual's subjectivity causes clinicians to face pressure in meeting aesthetic expectations and achieve what is often referred to as a ‘perfect smile'.^5^

Discolouration can be attributed to various factors, broadly categorised as either intrinsic or extrinsic. Intrinsic discolouration is further classified into either eruptive causes, including metabolic or genetic disorders, or post-eruptive causes, which are due to dental conditions, such as dental caries and tooth wear, or dental materials, like amalgam and intracanal medicaments.^6^ Localised intrinsic tooth discolouration is usually confined to a single tooth, commonly caused by trauma and tooth resorption. Moreover, generalised intrinsic tooth discolouration is caused by systemic conditions, such as amelogenesis and dentinogenesis imperfecta.^6^ Extrinsic discolouration could be generally caused by factors such as diet, habits and medications.^6^ There exists a range of methods to mask a discoloured tooth and selecting an appropriate approach is detrimental to the success of the treatment. This review aims to broadly identify the aetiology of a single tooth discolouration and clarify which minimally invasive treatment option is the most appropriate for the condition identified.

## Literature search

The search of the literature was conducted using various electronic databases, namely PubMed, Embase, Scopus and Science Direct from 1970-2024. The search terms used to yield results were outlined in Table 1. The articles obtained were categorised into history and importance of aesthetics. Other classifications were discolouration and its types, and treatment solutions for masking discoloured teeth. The criteria for inclusion and exclusion are outlined in Table 2.

**Table 1:** Search strategies

**Database**	**Search strategies**	**Results**
PubMed	‘Tooth discolouration' AND ‘treatment'	200
Science Direct	‘Tooth discolouration' AND ‘treatment'	214
Embase	‘Tooth discolouration' AND ‘treatment'	104
Scopus	‘Tooth discolouration' AND ‘treatment'	102
**Total**		620
**After inclusion and exclusion**		94

**Table 2:** Inclusion and exclusion of literature

**Selection criteria**				
**Inclusion criteria**		**Exclusion criteria**		
Clinical studies (randomised controlled trial, cohort studies and case series)	Adult patients	*In vitro*, *in vivo* studies	Articles not in English	Primary teeth

## Discolouration

The source of discolouration is imperative and should always be established before formulating a treatment plan, as it can influence the desired treatment drastically. Moreover, patients who usually seek whitening, or any other form of restorations to mask their discolouration, are usually demanding and should be aware of the treatment goal and have realistic expectations.^5^ This can be thoroughly explained during the initial consultation. At that time, written consent should be obtained. Photographs and shade guides can also be used as a treatment aid. Box 1 details the steps to follow for obtaining a diagnosis.

Box 1 Diagnosis of discolouration

- Medical and dental history
  - Clinical examination
  - Sensibility test
  - Percussion and palpation test
  - History of root canal treatment
  - History of trauma
- Radiographic examination
  - Check quality of root canal treatment and/or restorations
  - Check for any lesions
- Shade match
- Photographs
- Distinguish between the type, severity and degree of discolouration

### Intrinsic discolouration

**Fig. 1:**
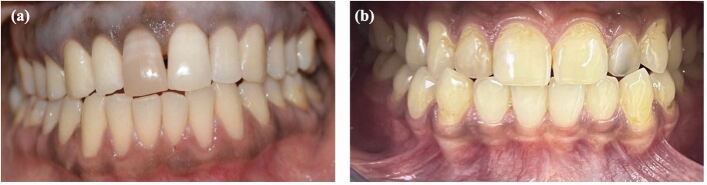
a) A single vital discoloured maxillary right central incisor with history of trauma. b) A single root canal-treated discoloured maxillary left lateral incisor

**Fig. 2:**
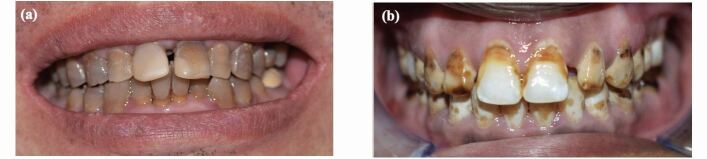
a, b) Patient is exhibiting clinical signs of generalised fluorosis

Intrinsic discolouration usually forms as a result of a change in the normal structural composition or thickness of the dental hard tissues and can be categorised into eruptive, post-eruptive, local and systemic causes.^7^ The local causes of intrinsic tooth discolouration are usually confined to a single tooth and are mainly due to endodontic origin or trauma (Fig. 1a and b). Systemic intrinsic discolouration is usually generalised and often emits a blue or pink colour that reflects through the enamel (Fig. 2a and b).^8^

### Extrinsic discolouration

Extrinsic discolouration refers to generalised staining that occurs on the outside surface of the tooth. Additionally, extrinsic discolouration can be classified into direct extrinsic discolouration and indirect extrinsic discolouration. It can also be classified as metallic and non-metallic extrinsic discolouration.^8^ Direct extrinsic discolouration occurs due to an influencing factor on the tooth surface, such as the chromogens that are derived from an individual's diet or exposure to the environment. Indirect extrinsic staining results from a chemical interaction of a compound with another compound. For instance, tooth staining was increased in individuals using a high amount of chlorhexidine.^9^ This is primarily due to the staining being associated with cationic antiseptics and metal salts. Similar to the direct extrinsic staining, non-metallic stains present due to habits, diets, environment, tobacco, medicaments and mouthrinses. Metallic stains arise due to occupational exposure and medications containing metal salts.^9^

### Internalised discolouration

A combination of both intrinsic and extrinsic discolouration is internalised discolouration that occurs in both the enamel and dentine surfaces. This is classified into developmental or acquired defects. The acquired defects are most commonly due to tooth wear and gingival recession, followed by dental caries and other restorative materials.^10^

## Treatment options

Various treatments are outlined to mask a discoloured tooth, as shown in Table 3. The various treatments aim at returning the tooth to its natural colour; however, this may not always be feasible. Restorative treatment arises in cases of extensive tooth destruction or severe discolouration, so options such as tooth preparations, different materials and cements are used to mask the discolouration.^11^

**Table 3:** Management of a discoloured tooth

**Minimally invasive**	**Invasive**
Scaling, polishing and air polishing	Indirect composite veneers
Microabrasion, air abrasion, megabrasion	Indirect ceramic veneers
Bleaching Vital Non-vital	Full coverage crowns
Direct composite veneers	

### Monitoring discolouration

The most conservative way to treat a discoloured tooth is to follow-up and monitor the tooth. In cases of pink discolouration that is present after injury, this can be reversible and the discolouration can resolve within 2-3 months.^12^

### Scaling, polishing and air polishing

Scaling, polishing and air polishing is beneficial in a discoloured tooth since it eliminates the extrinsic staining present. Air polishing can assist in removing extrinsic stains.^8^ The air-polishing handpiece uses a compound (either sodium bicarbonate, calcium carbonate, or aluminium tri-hydrate) which will be expelled through a mixing nozzle and placed against the tooth.^13^

### Bleaching

**Fig. 3:**
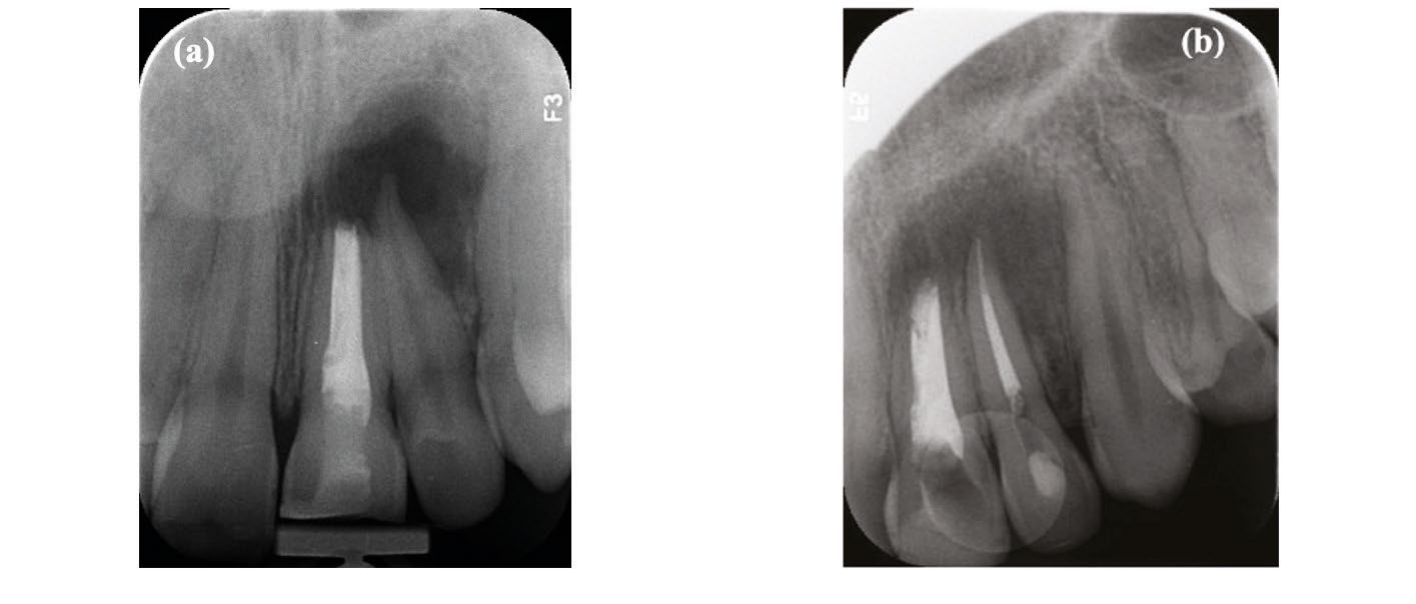
a) At the consultation visit, the patient complained of a discoloured tooth but had no pain. A pre-operative radiograph was taken as routine examination before commencing bleaching which shows a lesion, highlighting the importance of radiographic examination for diagnosis. b) A post-operative radiograph after re-treatment of the left maxillary central incisor and root canal treatment of the left maxillary lateral incisor

Many individuals are concerned with treating discolouration by using whitening products. The evidence is based on an increase in the sale of over-the-counter (OTC) teeth whitening products.^14^^,^^15^ Many patients seek cosmetic improvement to their teeth during in-office visits. But before any whitening products are applied to the dentition, it is crucial to do a full in-depth analysis of the teeth, as well as understanding the aetiology of the discolouration (Fig. 3a and 3b). Also, the vitality of the pulp and endodontically treated teeth will determine the appropriate management. Treatment options include:

- Vital tooth bleaching (external bleaching)
- Non-vital bleaching (walking bleach technique, inside-outside technique, in-office bleaching).

#### Vital tooth bleaching

Vital tooth whitening is a widespread method that is used by individuals to whiten their teeth. It can be used to eliminate yellow and brown hypoplastic discolouration which may be due to trauma.^16^ Vital tooth bleaching can also be used to manage the discolouration present of a single tooth due to canal obliteration or calcific metamorphosis.^17^

The approaches include: 1) in-office bleaching; 2) at-home or dentist-supervised nightguard; and 3) bleaching with OTC products.

In-office bleaching can only be administered by a professional dentist after the protection of the soft tissues by rubber dam or alternatives.^18^ In accordance with the General Dental Council^19^ (Cosmetics Products Enforcement Regulations 2013), bleaching products containing between 0.1−6% of hydrogen peroxide (equivalent to 20% carbamide peroxide) should be used after an appropriate clinical examination is carried out, exposure is limited to ensure intended use only, and the products should only be available through a dentist, dental hygienist, dental therapist or clinical dental technician.

Supervised nightguard whitening is considered the gold-standard technique and is often prescribed by dentists.^20^ It involves the use of 10% carbamide peroxide gel or 6% hydrogen peroxide in a tray and worn overnight for 2-6 weeks.^21^ The low concentration of the carbamide peroxide gel is beneficial since studies have shown that this decreases sensitivity and gingival inflammation and irritation that may occur from higher concentrations.^22^ This technique offers many advantages, such as self-administration by the patient, less chair time, a high degree of safety, fewer adverse effects and less cost. However, this technique requires high patient compliance which therefore could result in high relapse rates.^23^ Also, Alqahtani^20^ reported that, alternatively to low patient compliance, excessive use by patients was observed, resulting in frequent thermal sensitivity.

OTC products are applied without professional supervision and have been gaining popularity in recent years. The products include plastic strips which fit on the labial/buccal surfaces of teeth, whitening gels carried in disposable plastic trays, or toothpastes or mouthrinses.^21^ The active agents present in OTC products are carbamide peroxide or hydrogen peroxide; however, the General Dental Council^19^ states it is illegal for tooth-whitening products which contain more than 0.1% or less than 6% hydrogen peroxide to be supplied or administered for cosmetic purposes and only sold to dental practitioners, making the use of products containing a concentration of less than 0.1% insufficient to achieve a desired result. Swift *et al.*^24^ conducted a randomised, double-blind, placebo-controlled trial on 40 adults to evaluate the safety of 6% hydrogen peroxide whitening strips used twice daily for a period of six weeks. It resulted in teeth becoming lighter and less stained in comparison to the baseline and the placebo.

A clinical study by Wiegand *et al.*^25^ evaluated the 12-month stability of bleaching with different agents, such as 6% hydrogen peroxide strips, 15% carbamide peroxide gel, or 38% hydrogen peroxide gel. A decrease in the whitening effect was observed, concluding that colour is not stable in regard to lightness. However, even after 12 months, the colour change still did not achieve baseline values of unbleached samples.

#### Non-vital tooth bleaching

This approach includes: 1) the walking bleach technique; 2) the inside-outside technique; 3) in-office bleaching (light-activated); and 4) thermocatalytic bleach. Walking bleaching can use a variety of concentrations to achieve an appropriate result, such as sodium perborate and water (hydrogen peroxide), or sodium percarbonate, as well as carbamide peroxide or hydrogen peroxide gels.^8^ The mixture of sodium perborate and water is sealed into the pulp chamber of the affected tooth and it is repeated until the desired effect is reached. Moreover, the modified walking bleaching technique is done by using 30% hydrogen peroxide and sodium perborate sealed into the pulp chamber for one week;^26^ however, this technique is not commonly used due to concerns of invasive cervical resorption.^9^ According to Dahl,^22^ the patient is advised to return in 3-7 days to have more bleaching material placed and a total of 1-4 procedures may be required until the tooth is slightly lighter than the adjacent teeth.

**Fig. 4:**
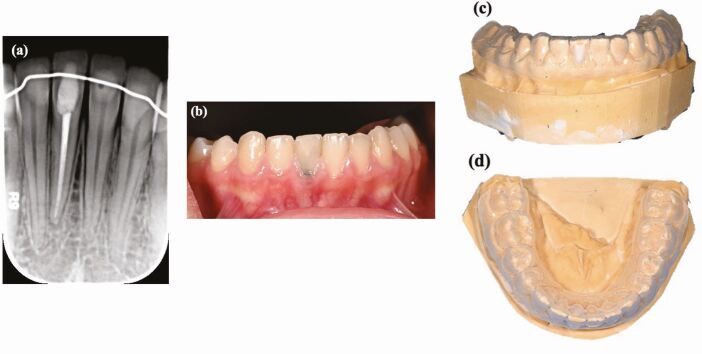
a) A pre-operative radiograph of a root canal-treated right mandibular central incisor. b) A pre-operative photo showing an endodontically treated tooth discolouration. The tooth was classified as C2 using the VITA Classical Shade Guide. c, d) A custom single tooth tray was fabricated and the tooth was managed using inside-outside bleaching using 10-15% carbamide peroxide in the bleaching tray every 4-6 hours

During inside-outside bleaching, 10-15% of carbamide peroxide is used in a bleaching tray. The palatal surface of the tooth will have an opening, where the access cavity is, so that the patient can place the bleaching agent inside. There will be an apical barrier of glass ionomer (GIC) placed by the dentist to maintain the coronal seal while also protecting the periodontium. This technique does require the patient's cooperation (Fig. 4).^27^ The patient regularly applies the bleaching agent with a syringe and externally on a tray every 4-6 hours. The patient is then reviewed after 2-3 days of application to assess any changes in the discolouration.^9^ In cases of inside-outside bleaching, Steiner *et al.*^28^ suggested the GIC barrier should be 2 mm thick and scalloped like a ‘bobsled tunnel' to reflect the contour of the cementoenamel junction as the inflammatory process leading to cervical invasive resorption may be initiated by the bleaching agent reaching the periodontium. Moreover, Amato *et al.*^29^ conducted a study on patients with a single endodontically treated incisor subjected to both in-office intracoronal dental bleaching and the walking bleach technique with 10% carbamide peroxide gel. The patients were recalled at a follow-up of 12-month intervals for 25 years. An 85% success rate was observed, demonstrating that 10% carbamide peroxide proved to be effective as a long-lasting treatment.

In-office bleaching produces short-term results and is not favoured for the management of a single tooth discolouration and this is mainly due to tooth dehydration that occurs.^8^^,^^30^ This procedure is performed using 35% hydrogen peroxide that is applied onto the tooth and the access cavity.^31^

A clinical study by Knezevic *et al.*^32^ aimed to establish the results of bleaching endodontically treated teeth using various approaches, as well as the effects of etiological factors and the time relapse after the endodontic treatment. This study confirms that there was no statistical significance between the success and the underlying etiological factor of the discolouration. Furthermore, tooth bleaching was more efficient in the teeth that were recently endodontically treated than teeth that were treated more than five years before bleach.

The thermocatalytic technique is similar to the conventional technique: 30-35% hydrogen peroxide is placed in the pulp chamber and activated via heat application with electric heating devices in order to accelerate the release of reactive oxygen species and increase the bleaching effect of endodontically treated teeth.^33^ However, there is an increased risk of cervical root resorption when this technique is applied and therefore it is currently not advisable.^30^

#### Complications associated with bleaching

Despite the extensive knowledge reported about bleaching, several issues remain associated with their use. These issues include tooth hypersensitivity, resorption, gingival irritation, modifications in surface morphology and effects on restorative materials.^34^

Tooth sensitivity is the most commonly reported side effect from bleaching. Whether performed at home or in-office, studies indicate that between 18−78% of patients experience sensitivity during their bleaching treatments.^26^ A systematic review by de Geus *et al.*^35^ evaluated the risk and intensity of tooth sensitivity during in-office or at-home bleaching. The bleaching protocol for at-home bleaching included carbamide peroxide at different concentrations, ranging from 15-32% across nine studies. In-office bleaching protocols predominately used 35% hydrogen peroxidel; however, some studies used concentrations of 38% or 25%. This study concluded no significant difference in the risk or intensity of tooth sensitivity observed when comparing in-office or at-home bleaching; however, variations, such as daily use time and number of bleaching sessions, were not taken into consideration. Additionally, in-office bleaching of non-vital teeth have been linked to root resorption due to use of high concentrations of hydrogen peroxide and is no longer recommended.^31^

The most common complication reported in the literature that can arise with non-vital bleaching is external cervical root resorption (ECR). It is believed this complication results from when the bleaching agent seeps through the dentinal tubules, causing necrosis of the cementum, leading to inflammation of the periodontal ligament and resulting in ECR.^36^^,^^37^ Some of the several risks highlighted that could result in ECR include use of high concentration of hydrogen peroxide, application of heat, orthodontic treatment, history of trauma, presence of bacteria and no barrier present over the gutta percha.^36^^,^^37^^,^^38^ Fortunately, in recent years, there has been a reduction in the incidence rates of ECR caused by bleaching. This can be attributed to the standardisation of bleaching protocols and improvements in the agents used.^36^^,^^39^

Relapse of discolouration in non-vital teeth following bleaching is relatively common. It may occur over a period of time (20% at three years and one-third of cases after 16 years).^40^ Other studies report rates of 10% after two years, 20-25% after five years and 49% after eight years.^8^

### Abrasion

Microabrasion can be used to eradicate discolouration that occurs within enamel and intrinsic stains, such as fluorosis.^8^ Microabrasion erodes the surface layer of enamel, resulting in a smooth and prismless layer.

Air abrasion has been explained as a pseudo-mechanical, non-rotary method of cutting dental hard tissues, using kinetic energy of a stream of desiccated abrasive particles to bombard the tooth surface at a high velocity.^40^ The particles used for air abrasion are alumina particles. Extrinsic stains respond well to air abrasion; however, this is not a conservative method in eliminating discolouration. Subcutaneous emphysema - a rare complication - has been reported in the literature when using air-driven instruments. Some of the causes proposed are the combination of an air-polishing device with an abrasive powder from a different manufacturer which may alter the speed of the microparticles increasing the abrasive impact on the teeth.^38^ Air-polishing devices should be used cautiously, while limiting their use to 5-10 seconds per tooth with overlapping strokes and the use of rubber dam application during these procedures to reduce the risk of epithelial erosion.^41^^,^^42^

Mega-abrasion is beneficial when removing stains that are yellow or brown and are present on enamel. The procedure removes the stains by mechanical properties and a placement of composite resin is then required.^16^

### Resin infiltration

This product was initially designed to treat incipient interproximal caries and anterior white spot lesions by etching the tooth with 15% hydrochloric acid, drying with an ethanol solution, and applying a triethylene glycol dimethacrylate-based resin infiltrate (ICON).^43^

In current clinical evidence, clinical experience with this technique has proven to be effective in masking discolouration of both decalcification lesions and teeth with developmental non-carious aetiology.^44^

A clinical trial has reported better masking effects in relation to cases of fluorosis than hypomineralisation; however, a thicker discolouration that is visually apparent will be difficult to infiltrate and mask.^45^

## Restorative treatment options

Restorative treatment outcomes are often preferred due to their high aesthetic outcomes. Direct and indirect restorations may also be used to mask dark discolouration that may be present on the tooth surface.

### Direct restorations

Composite restorations may also be used to mask discolouration. When using composites in a Class IV restoration, the incisal edges can be bevelled, providing a pleasing aesthetic result. Another advantage is the single-visit appointment for patients suffering from trauma, which can also be assisted by the prefabricated celluloid shells to restore the anatomy.^46^ One of the disadvantages of composite is its discolouration over time and this could be a potential challenge when attempting to mask a discoloured tooth.

The different enamel and dentine shades can mask the underlying discolouration up to an extent. If a dark discolouration is present, then a layer of resin-based opaquer can be placed initially over the discoloured areas.^47^ The composites can also be layered with various shades, which is beneficial when the underlying substrate is a highly discoloured tooth. The darkness of the oral cavity also creates a grey illusion which leads to an unsatisfactory colour match. All these factors should be considered when attempting to mask a discoloured tooth, since the human eye does not possess high potential in colour matching.^48^^,^^49^^,^^50^

Kim *et al.*^51^ successfully masked a single tooth by using a range of 0.45-2.00 mm layers of composite resin against a C4 dark background (VITA Classical Shade Guide). The study used a 0.5-1.0 mm of composite resin to mask discolouration of a C4 tooth shade and stated that 1.0-2.00 mm was satisfactory to mask the background colour.

The success of direct restorative techniques is congruent on the operator's ability to accurately restore the shape, contour, and above all, the natural colour of the tooth.^1^ Direct composite restorations have demonstrated clinical longevity, with survival rates exceeding 80% after five years.^2^ According to Coelho *et al.,*^33^ the annual failure rate was reported at 4.9% for vital teeth and 9.8% for non-vital teeth, emphasising the differing outcomes based on tooth vitality.

### Simulation

Simulation is also a technique which has been proposed by Setein *et al.*^52^ to mask discolouration but is not widely used. The technique involves the use of composite resin to mimic discolouration. Patients would like to conceal the discolouration and not highlight it, especially if the discolouration is of a dark shade. On the other hand, Setein *et al.*^52^ highlighted this technique in a case of patients presenting with yellow discolouration to their upper incisor.

## Conclusion

**Fig. 5:**
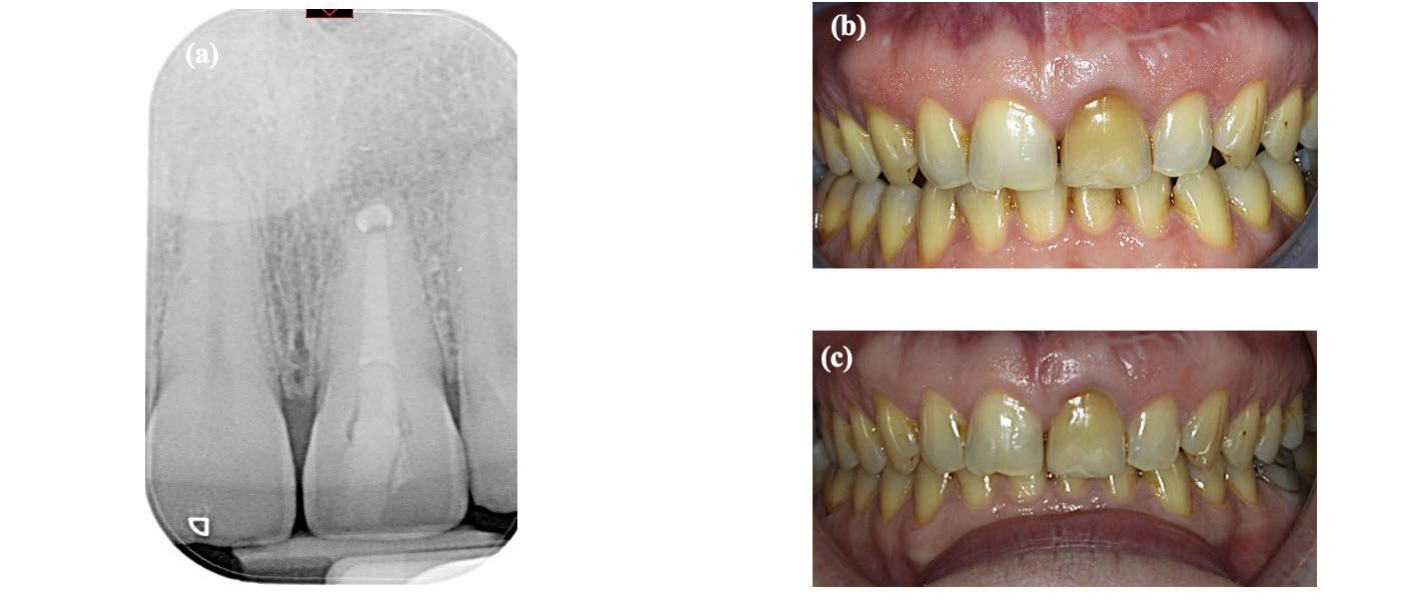
a) A periapical radiograph of a root canal-treated left maxillary central incisor. b) A pre-operative photo showing an endodontically treated tooth discolouration. The tooth was classified as C4 using the VITA Classical Shade Guide. c) The tooth was managed using inside-outside bleaching using 10-15% carbamide peroxide in a bleaching tray; however, the discolouration was still present and the tooth was indicated for veneers

The importance of aesthetics is constantly increasing in today's society, especially in the field and scope of dental aesthetics. This initial review (the first part in a two-part series) has provided a comprehensive outline of the aetiology of tooth discolouration and examines the considerations regarding whether a minimally invasive procedure might be the optimal choice. In the management of a discoloured tooth, it is important to obtain a complete dental history along with clinical and radiographic examination to confirm the aetiology and diagnosis. Among the management discussed, a minimally invasive procedure, such as abrasion, can be used in cases of fluorosis or mild discolouration present on enamel. Bleaching provides an optimal treatment as it can successfully treat discoloured teeth with minimal side effects, particularly in cases of endodontically treated teeth. If a tooth is still discoloured after several sessions of bleaching, a direct composite veneer which requires no preparation or minimal preparation can be done (Fig. 5). However, if more challenges emerge during treatment, then a more invasion option is the best approach.

## Data Availability

The data analysed in this review are available from the corresponding author on request.
